# Mobile Apps for COVID-19 Detection and Diagnosis for Future Pandemic Control: Multidimensional Systematic Review

**DOI:** 10.2196/44406

**Published:** 2024-02-22

**Authors:** Mehdi Gheisari, Mustafa Ghaderzadeh, Huxiong Li, Tania Taami, Christian Fernández-Campusano, Hamidreza Sadeghsalehi, Aaqif Afzaal Abbasi

**Affiliations:** 1 Institute of Artificial Intelligence Shaoxing University Shaoxing China; 2 Department of Computer Science and Engineering, Saveetha School of Engineering Saveetha Institute of Medical and Technical Sciences Chennai India; 3 School of Nursing and Health Sciences of Boukan Urmia University of Medical Sciences Urmia Iran; 4 Florida State University Tallahassee, FL United States; 5 Departamento de Ingeniería Eléctrica Facultad de Ingeniería Universidad de Santiago de Chile Santiago Chile; 6 Department of Neuroscience Faculty of Advanced Technologies in Medicine Tehran Iran; 7 Department of Earth and Marine Sciences University of Palermo Palermo Italy

**Keywords:** COVID-19, detection, diagnosis, internet of things, cloud computing, mobile applications, mobile app, mobile apps, artificial intelligence: AI, mobile phone, smartphone

## Abstract

**Background:**

In the modern world, mobile apps are essential for human advancement, and pandemic control is no exception. The use of mobile apps and technology for the detection and diagnosis of COVID-19 has been the subject of numerous investigations, although no thorough analysis of COVID-19 pandemic prevention has been conducted using mobile apps, creating a gap.

**Objective:**

With the intention of helping software companies and clinical researchers, this study provides comprehensive information regarding the different fields in which mobile apps were used to diagnose COVID-19 during the pandemic.

**Methods:**

In this systematic review, 535 studies were found after searching 5 major research databases (ScienceDirect, Scopus, PubMed, Web of Science, and IEEE). Of these, only 42 (7.9%) studies concerned with diagnosing and detecting COVID-19 were chosen after applying inclusion and exclusion criteria using the PRISMA (Preferred Reporting Items for Systematic Reviews and Meta-Analyses) protocol.

**Results:**

Mobile apps were categorized into 6 areas based on the content of these 42 studies: contact tracing, data gathering, data visualization, artificial intelligence (AI)–based diagnosis, rule- and guideline-based diagnosis, and data transformation. Patients with COVID-19 were identified via mobile apps using a variety of clinical, geographic, demographic, radiological, serological, and laboratory data. Most studies concentrated on using AI methods to identify people who might have COVID-19. Additionally, symptoms, cough sounds, and radiological images were used more frequently compared to other data types. Deep learning techniques, such as convolutional neural networks, performed comparatively better in the processing of health care data than other types of AI techniques, which improved the diagnosis of COVID-19.

**Conclusions:**

Mobile apps could soon play a significant role as a powerful tool for data collection, epidemic health data analysis, and the early identification of suspected cases. These technologies can work with the internet of things, cloud storage, 5th-generation technology, and cloud computing. Processing pipelines can be moved to mobile device processing cores using new deep learning methods, such as lightweight neural networks. In the event of future pandemics, mobile apps will play a critical role in rapid diagnosis using various image data and clinical symptoms. Consequently, the rapid diagnosis of these diseases can improve the management of their effects and obtain excellent results in treating patients.

## Introduction

Following the widespread and rapid outbreak of COVID-19, the disease crossed geographical borders and had a devastating impact on the health, economy, and well-being of the worldwide population. According to the World Health Organization (WHO), high-severity COVID-19 was reported in 16%-21% of patients and almost 3% died. In the case of other variants, however, local statistics in many countries indicated a high mortality rate, with some studies estimating a mortality rate of 4% or higher. Due to the novelty of the disease, ways to deal with it were not known early on; still, researchers considered the screening and rapid diagnosis of patients and their separation from healthy people to be significant steps in fighting the disease [1,2].

Early and low-cost diagnosis of infections in any pandemic is essential for pandemic control. Therefore, if it is possible to diagnose and quarantine infected cases in the earliest phases of an outbreak, the outbreak can be managed in the epidemic phase and will not become a pandemic. In the COVID-19 pandemic, various diagnostic methods have been used, with the polymerase chain reaction (PCR) test being the primary diagnostic tool. Nevertheless, PCR is a time-consuming and costly method; until being diagnosed with this diagnostic test, patients might freely transmit COVID-19 and accelerate its conversion into a pandemic by increasing the transmission rate [3,4].

In the COVID-19 pandemic, a wide range of technologies came to aid in the faster diagnosis and screening of people with infection, many of which proved successful. Meanwhile, mobile phones, as highly abundant tools and an information gateway, helped people update their information and make more accurate decisions. In addition to being a platform for installing essential and valuable apps to detect people with infection, smartphones help track people and examine the keywords used by them for making a diagnosis [5-7].

Numerous technical and review studies have addressed smartphone apps for COVID-19 management. Many of these studies have dealt with various mobile apps used to estimate the prevalence, lessons, opportunities, and challenges of these devices and disease management. Still, none of them has adequately focused on detection and diagnosis. This study is the first to systematically review all the studies that have used smartphone technology to detect and diagnose COVID-19. Previous reviews have examined a limited number of studies. Herein, by covering the maximum number of databases, an effort was made to explore all published papers that used smartphones to diagnose COVID-19. In the area of mobile apps for the diagnosis of COVID-19, a thorough and complete study has not been conducted. Alnazi [8] examined apps related to COVID-19 released on Google Play. The 12 studies reviewed included mobile-based software for contact tracing, awareness building, appointment booking, and online consultation. The author only reviewed free apps, however, and although this study was conducted in 2021, it did not include many scientific or commercial apps [8].

Asadzadeh et al [9] determined the scope of mobile solutions in the COVID-19 pandemic and reviewed 16 mobile app studies on COVID-19–related data processing. The reviewed studies were classified into 4 categories: prevention, diagnosis, treatment, and protection. Despite noting a different range of mobile apps, this study did not mention the details and algorithms of these studies [9].

Aslani et al [10] studied mobile health apps in pandemics and epidemics. They examined 17 studies and explored common respiratory diseases and lung infections. Although this study was published during the COVID-19 pandemic, it did not mention mobile apps related to COVID-19 [10].

Kondylakis et al [11] examined 12 studies on mobile apps for COVID-19 data analysis in a more comprehensive investigation. These 12 studies covered the following domains: training, information sharing, risk assessment, self-management of symptoms, contact tracing, home monitoring, and decision-making. Still, this study did not include studies using machine learning (ML) methods to predict and diagnose COVID-19. It also did not deal with mobile apps for COVID-19 diagnosis and primarily focused on studies on COVID-19 education, care, and management using mobile apps [11].

Almalki et al [12] analyzed and discussed all the apps available on Google Play and the Apple Store and provided a brief explanation. Among its flaws, this study did not review scientific or academic studies and only examined mobile apps available in the market, many of which were developed without scientific or clinical supervision. Therefore, it is difficult to rely on these apps, as they lack scientific support and cannot be introduced to or proposed by the communities [12].

Table 1 lists some studies that have addressed mobile apps for COVID-19 data management, including the first author’s name, the country, the main topic, and the number of studies covered.

**Table 1:** Related studies on mobile apps for detecting and diagnosing COVID-19.

Author and country	Main topic	Studies covered, n
Alanzi [8], Saudi Arabia	Mobile app used during COVID-19	12
Asadzadeh et al [9], Iran	Mobile health solutions	16
Aslani et al [10], Iran	Mobile health apps for epidemic and pandemic outbreaks	17
Kondylakis et al [11], Greece	Mobile app for COVID-19	12
Almalki et al [12], Saudi Arabia	Implemented an app to combat COVID-19	115

This study aimed to fill the gap left by previous reviews by conducting a comprehensive review of studies on smartphone apps for the diagnosis of COVID-19, providing solutions based on technological models, and answering research questions so that researchers and health systems can envision devices and their apps in preventing future pandemics.

## Methods

### Search Criteria

This systematic review, which was conducted for the first time using this method, aimed to identify relevant studies related to detecting and diagnosing COVID-19 using a variety of smartphone apps. The systematic search strategy was developed based on previous studies and the authors’ knowledge. The main objective was to address the following analytical questions (AQs):

- AQ1: What are the uses of smartphones for COVID-19 detection and diagnosis?
- AQ2: What data do smartphones use to detect and diagnose COVID-19?
- AQ3: Which artificial intelligence (AI) methods and algorithms are used to process smartphone data?
- AQ4: How successful have smartphone apps been in COVID-19 detection and classification?
- AQ5: What suggestions can be made to improve the quality of mobile apps in disease diagnosis and pandemic control?

We reviewed electronic databases publishing papers on medicine and computer science. We concluded that PubMed, Web of Science (WoS), Scopus, IEEE, and ScienceDirect contain the most relevant papers. The search used the following keywords and logical expressions: ((“COVID-19”) AND (Detection OR Diagnosis) AND (Smartphone OR Mobile Application OR Mobile App)). The investigation was conducted from November 1, 2019, to late April 2022, and relevant published papers were extracted. The Embase database was eliminated from the examination due to the proximity of the publications.

### Data Extraction

**Figure 1:**
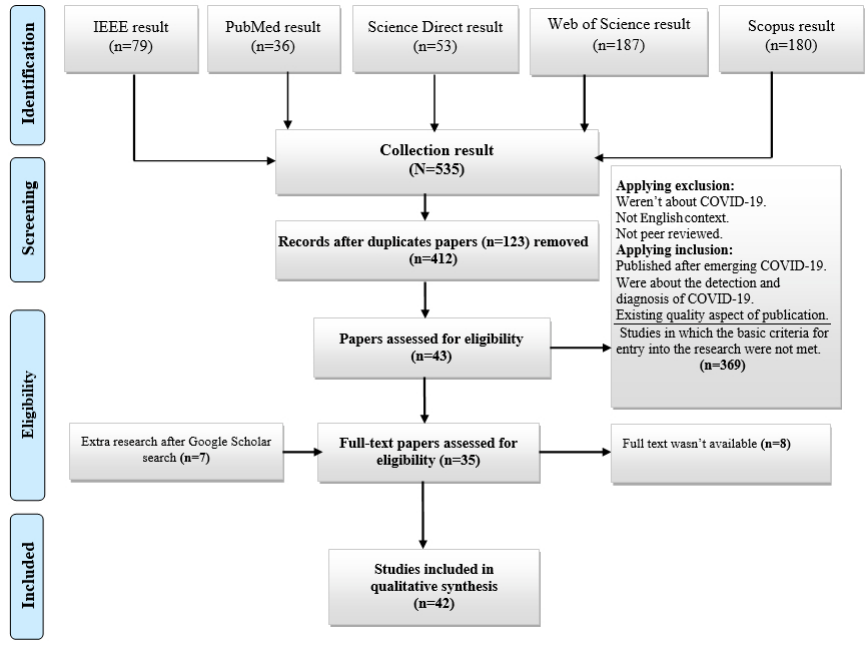
The review process and how to exclude papers according to the PRISMA flow diagram. PRISMA: Preferred Reporting Items for Systematic Reviews and Meta-Analyses.

Relevant studies and the main elements of their methodology and results were recorded in data extraction forms in order to identify AI algorithms and techniques. Two researchers (authors AMR and MG) performed data extraction, and discrepancies between the researchers were resolved by discussion with an independent researcher (author AH). The extracted data elements included the first author’s name, country of origin, research population, data used, purpose, method, the role of the mobile app, and the evaluation method. The search in reputable databases was performed based on the search strategy, and 535 papers were extracted. After reviewing the papers’ abstracts and full texts, applying the inclusion and exclusion criteria, and selecting papers relevant to the title of this study, 42 (7.9%) full-text papers were finally selected. This process was performed based on the PRISMA (Preferred Reporting Items for Systematic Reviews and Meta-Analyses) flowchart, as shown in Figure 1.

## Results

### Study Details

**Figure 2:**
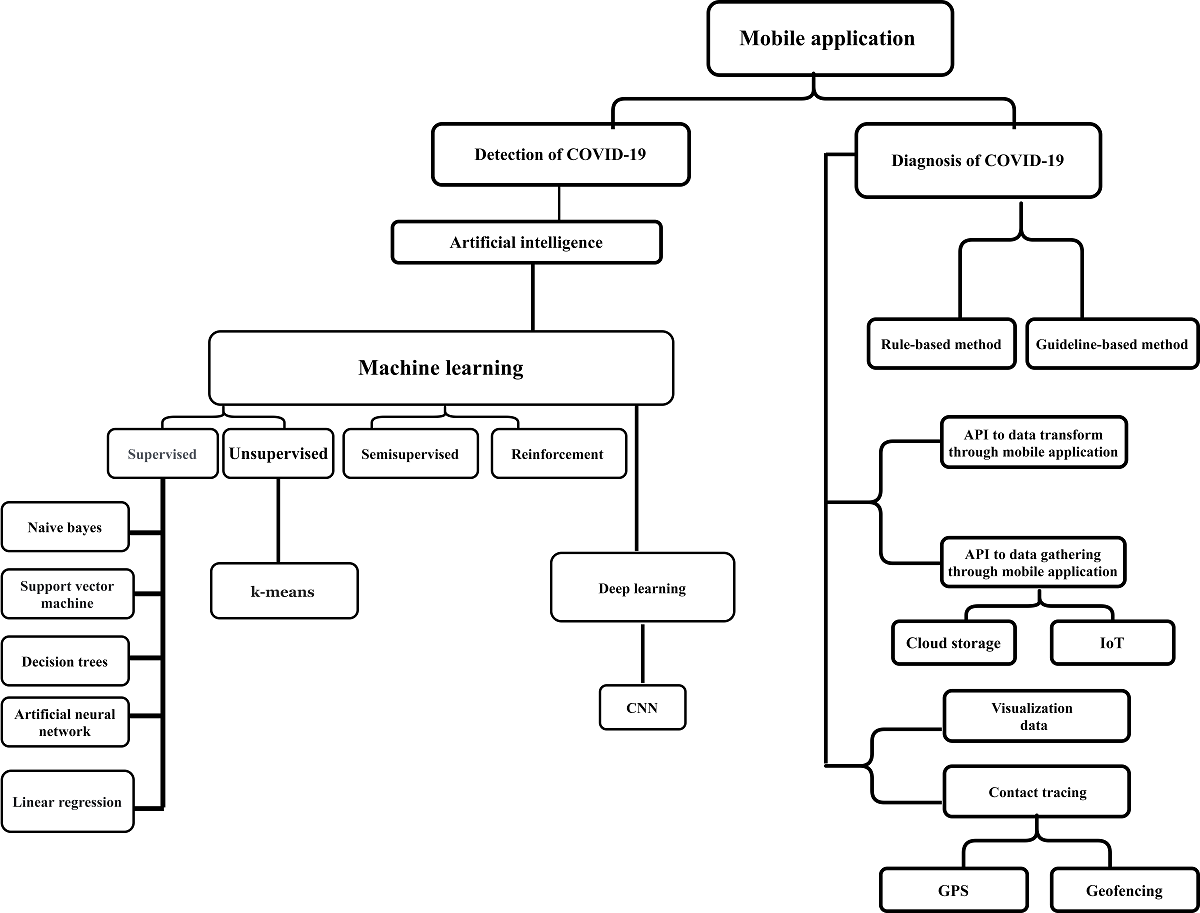
Taxonomy of mobile applications for the COVID-19 pandemic. API: application programming interface; CNN: convolutional neural network; IoT: internet of things.

Due to the newly emergent status of COVID-19, the titles, abstracts, and keywords of all the papers published between 2020 and 2022 were reviewed, and 42 (7.9%) of 535 papers were ultimately selected as eligible. By comprehensively examining the mobile apps, we found that the role of smartphones was described in 6 areas with different types of COVID-19 data sets, including “Smartphones play the role of a platform for data collection,” “visualizing the input data,” “installing AI-based processing software,” “determining contact tracing,” and “COVID-19 data processing based on role-based and guideline-based methods,” to detect and diagnose COVID-19. A taxonomy was developed to better organize the content and concepts related to mobile apps for COVID-19 diagnosis and detection, as shown in Figure 2.

The taxonomy presented in this research classified the studies conducted in line with the investigation into 2 branches: detection and diagnosis. In the field of detection, the AI method was used more, which included traditional ML algorithms, such as supervised, unsupervised, semisupervised, and reinforcement learning; however, deep learning (DL) dealt with images and sounds. In the field of diagnosis, rule- and guideline-based techniques were mainly used to diagnose COVID-19 with mobile apps.

### Detection vs Diagnosis of COVID-19 Using Mobile Apps

The terms “detection” and “diagnosis” were used interchangeably in many of the studies, but they differed in meaning and usage. These 2 terms are used differently in different settings. In clinical settings and diagnostic laboratories, the term “diagnosis” is used, while in computer vision and pattern recognition, observing the first definite signs in determining the status of a disease is called “detection.” By examining the differences between these 2 terms in the dictionary and by inquiring from clinicians, a 2021 study found that detection identifies diseases in a set of patient and nonpatient cases. In detection, the disease is distinguished from other conditions, such that other cases may or may not have a disease-free status. However, the precise level and type of disease are completely specified during diagnosis. In the diagnosis concept, different cases might have a disease status or belong to other classes of abnormality or nonhealth, which can be determined [4,13-17].

**Figure 3:**
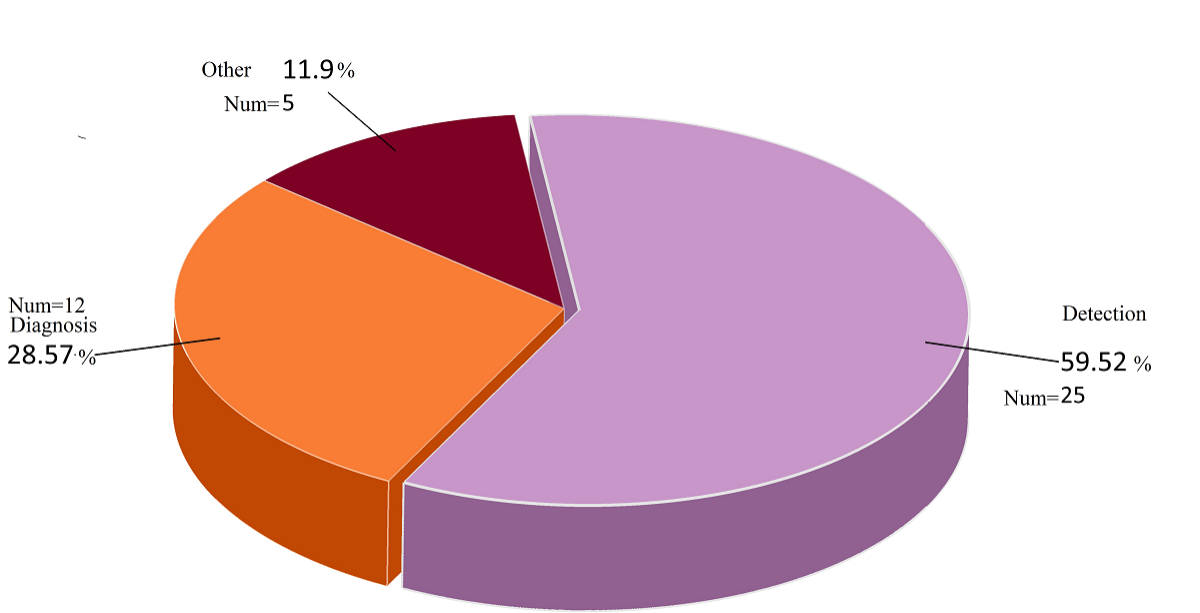
Aim of studies on the analysis of COVID-19 data in mobile applications.

Accordingly, detection was used for studies that distinguished cases of COVID-19 from healthy and normal cases. In contrast, diagnosis was used for studies that distinguished COVID-19 from other infectious pulmonary diseases (eg, different types of pneumonia). Detection makes sense in sets where other conditions (not infected with COVID-19) are specified, and COVID-19 can be distinguished with certainty from types of pneumonia or other coronaviruses [4,13,14]. By reviewing the 42 extracted papers, we found that 25 (59.5%) papers used DL to detect (identify) COVID-19, whereas 12 (28.6%) studies used it to diagnose and distinguish COVID-19 from other diseases. In addition, 3 (7.1%) studies did not precisely mention that diagnosis was their objective, while in 2 (4.8%) studies, the goal was to diagnose and detect. Figure 3 shows the amount of research performed to detect and diagnose COVID-19.

### Overview of the Role of Smartphone Apps in the Diagnosis of COVID-19

The apps were classified into the following 6 categories by reviewing all the studies conducted on mobile technologies and apps in line with our research objectives: contact tracing, data gathering, data visualization, AI-based diagnosis, rule- and guideline-based diagnosis, and data transformation.

#### Mobile Apps for Contact-Tracing Analysis

One of the main uses of apps during the COVID-19 pandemic was for contact tracing to diagnose and classify COVID-19. Thus, smartphones were used to diagnose patients with COVID-19 who had been in contact with people with infection. Many of the studies used mobile apps to trace people via trackers, geofencing, and GPS.

#### Mobile Apps for Gathering Data From Users

The sole purpose of some mobile apps was to collect data for processing. These apps received data through a standard smartphone from mobile devices as input devices in perception layers and sent them to the edge and cloud layers, where processing took place. In this case, the mobile apps sent data from the output of biosensors to higher-level systems for processing.

#### Mobile Apps for Data Visualization

Mobile apps were used to visualize the data received from biosensors or other data collection tools in many of the studies. After data were received, they were displayed to users via statistical charts. Smartphones carried out this task as the first step in data processing, which means gathering data and visualizing these data for COVID-19 diagnosis. In the next stage, these data were transferred to higher levels of processing, such as cloud and fog spaces.

#### Mobile Apps Used to Analyze COVID-19 Data Based on AI Algorithms

One of the most frequently used mobile apps for COVID-19 diagnosis was an AI-based application in which learning occurred. Studies used different clinical data, sounds, and radiology images. Data were collected in 2 ways: by mobile app designers and developers and by clinical app users using mobile apps.

Since most mobile apps processed and predicted COVID-19 infection, they incorporated different AI algorithms that involved 2 types of methods: methods based on traditional ML algorithms and those based on DL algorithms.

#### Mobile Apps Used to Analyze COVID-19 Data Based on Guidelines and Rules

A notable study on COVID-19 diagnosis using mobile phones involved ontologies, clinical guidelines, and rules. Due to COVID-19 diagnostic guidelines in health care centers, guideline- and rule-based methods for COVID-19 diagnosis are expected to be popular among clinicians and physicians. By incorporating these guidelines and rules into mobile apps, clinicians and stakeholders can see the process as more tangible and acceptable.

#### Mobile Apps as a Platform to Transform Messages and Data

Another application of mobile phones for COVID-19 diagnosis was processing keywords related to COVID-19 in social media using smartphones or analog messaging methods. In this method of COVID-19 diagnosis, the main terms representing COVID-19 diagnosis were counted and the COVID-19 diagnosis was made based on the number of uses of these words and their relationship with pronouns and sentence components.

### Data Used in Mobile Apps for COVID-19 Detection and Diagnosis

A wide range of data were used in mobile apps, and according to specialty domains, different scientific disciplines made the initial diagnosis of COVID-19 differently. Mobile apps used a range of data for collection, classification, analysis, transfer, and visualization. These apps dealt with various data, including nasal swap samples, personal clinical data, signs and symptoms, voice and sounds, radiology images, and words and terms in media.

Several of the studies used a combination of data types. Some others used subjective clinical data and symptoms. Moreover, some apps used a variety of radiology images and patients’ voices (cough).

Table 2 lists 11 (26.2%) studies on mobile apps for diagnosing COVID-19. This table includes the study, data, the application method, the mobile role, and the evaluation output.

**Table 2:** Studies (n=11) evaluating mobile apps used for COVID-19 diagnosis (or a goal similar to diagnosis).

Study	Data	Goal	Method	Mobile role	Evaluation output
Bindra et al [18]	Symptoms, clinical and bibliography data	COVID-19 risk prediction	ML^a^	Platform for the applied model to calculate risk prediction	N/A^b^
Sharma et al [19]	Sound	Diagnosis	ML	Platform for analysis; speech and sound analysis	N/A
Nema et al [20]	Symptoms	Diagnosis	Rule-based reasoning	Gathering symptoms and receiving alerts (SMS)	N/A
Quer et al [21]	Smartwatch, activity tracker data, symptoms, testing results	Differentiating COVID-19–positive status	Single decision threshold	Data collection	Accuracy=83.3%
Elagan et al [22]	Heart rate, blood cell counts, temperature	Diagnosis	Sending patient data to a physician and receiving output from the physician	Estimating the heart rate, receiving data from wireless sensors used to measure white blood cells (WBCs) and red blood cells (RBCs), and estimating air temperature	N/A
Imran et al [23]	Cough sounds	Diagnosis	DL^c^	Receiving cough sounds and analyzing them using a designed app	N/A
Mukhtar et al [24]	Cough, SpO_2_^d^, temperature	Diagnosis	Rule-based reasoning	Collecting data, sending data, showing the assessment	N/A
Koshti et al [25]	Symptoms	Diagnosis	ML	App platform	Accuracy=99%
Ertuğrul et al [26]	Personal data, observed symptoms (images, sounds)	Prediction	Ontology and rules	App platform	N/A
Maghded et al [27]	CT^e^ scan, cough, voice, breath sounds, fatigue	Diagnosis	DL	App platform	N/A
Rangarajan and Ramachandran [28]	CT images	Diagnosis	DL	App platform	N/A

^a^ML: machine learning.

^b^N/A: not applicable.

^c^DL: deep learning.

^d^SpO_2_: saturation of peripheral oxygen.

^e^CT: computed tomography.

There are several mobile apps available to detect COVID-19, as shown in Table 3. An overview of 31 (73.8%) studies is provided in the table, which includes the study, data, the application method, the mobile role, and evaluation results.

**Table 3:** Studies (n=31) that used mobile technologies to detect COVID-19.

Study	Data	Goal	Method	Mobile role	Evaluation output
Gökcen et al [29]	Cough	Detection	DL^a^	Platform for applying a COVID-19 detector via cough sounds	Accuracy=79%; *F*_1_-score=80
Mao et al [30]	Wastewater sample	Detection	Biosensor analysis	Interface to send data	N/A^b^
Stasak et al [31]	Speech voice	Detection	ML^c^	App platform	Accuracy>82%-86%
Alkhodari and Khandoker [32]	Breath, cough, voice	Detection	DL	App platform	Accuracy=94.5% and 92.1%
Al-zubidi et al [33]	Blood index	Detection	ML	App platform	Accuracy=89%
Abdulrazaq Alshekhly et al [34]	Thermal images and location	Detection	Thermometer and Send location	API^d^ to calculate and send data	N/A
Berquedich et al [35]	Contact tracing	Detection and Management	Guideline based	App platform to prescribe drugs and send an alarm	N/A
Karataş et al [36]	Cough, voice	Detection	ML	App platform	Accuracy=96.5%
Awasthi et al [37]	Ultrasound images	Detection	DL	App platform	Accuracy=83%
Tawfik et al [38]	Cough sounds	Detection	ML and DL	App platform	Accuracy=98%
Krisnanik et al [39]	Symptoms	Detection	Rule-based reasoning	App platform	N/A
Ponomarchuk et al [40]	Breath and cough sounds	Detection	DL	App platform	N/A
Shreyas et al [41]	X-ray images	Detection	DL	App platform	Accuracy=98.4%
Mohsin et al [42]	Symptoms	Detection	Rule-based reasoning	App platform	N/A
Sanjeev et al [43]	Cough and clinical data (SpO_2_^e^ level, body temperature, heart rate, symptoms)	Detection	ML	App platform	Accuracy=85%
Ponomarchuk [40]	Breath and cough sounds	Detection	DL	App platform	N/A
Bushra et al [44]	X-ray images	Detection	DL	Platform to trace and analyze keywords	Accuracy=98.6%
Verde et al [45]	Cough	Detection	ML	Platform to analyze cough sounds	Accuracy=82%
Stanciu et al [46]	Bluetooth data	Virus Detection	Contact management	Contact tracing	N/A
Han et al [47]	Nasal swab sample	Virus Detection	Fluorescent aptasensors	Data visualization	N/A
Fozouni et al [48]	Nasal swab sample	Detection	RNA analysis	Data visualization	Sensitivity in less than 30 minutes
Coppock et al [49]	Audio and sound	Detection	ML	Platform to install an app	N/A
Wong et al [50]	Symptoms	Detection	Medical protocol	Receiving data and analysis using a designed app	N/A
Hijazi et al [51]	Heart rate, feeling features, blood pressure	Detection	ML	Collecting data from users	Mean accuracy= 83.3% (SD 1.6%)
Echeverría et al [52]	Sounds, symptoms	Early detection, management of close contacts	Guidelines	Gathering signs and symptoms	N/A
Verma et al [53]	CT^f^ scan	Detection	DL	Process unit and platform for applied model	Accuracy=99.6%; *F*_1_-score=99.6
Chen et al [54]	Spike protein, nucleocapsid protein	Detection	Data transfer	Receiving, gathering, and transmitting data in edge layers	N/A
Chen et al [55]	Temperature, heartbeat	Detection	Comparing received data with normal data	Receiving data from a sensor and sending it to the database	N/A
Wang et al [56]	Keywords on social media	Detecting the SARS-CoV-2 outbreak	Analysis of emerged keywords	Platform to install WeChat to trace and analyze keywords	N/A
Udhaya Sankar et al [57]	Speech, voice	Detection	Computational audit techniques	Data collection	N/A
Sun et al [58]	Horse nasal swab samples	Detection	ML	Smartphone-based collection and visualization of data	Results achieved in approx. 30 minutes

^a^DL: deep learning.

^b^N/A: not applicable.

^c^ML: machine learning.

^d^API: application programming interface.

^e^SpO_2_: saturation of peripheral oxygen.

^f^CT: computed tomography.

Having thoroughly scrutinized the particulars of the investigations delineated in Tables 2 and 3, the methodologies implemented therein, the scholarly community involved, and the significance of mobile apps in the detection of COVID-19, we were equipped to provide answers to the AQs.

**Figure 4:**
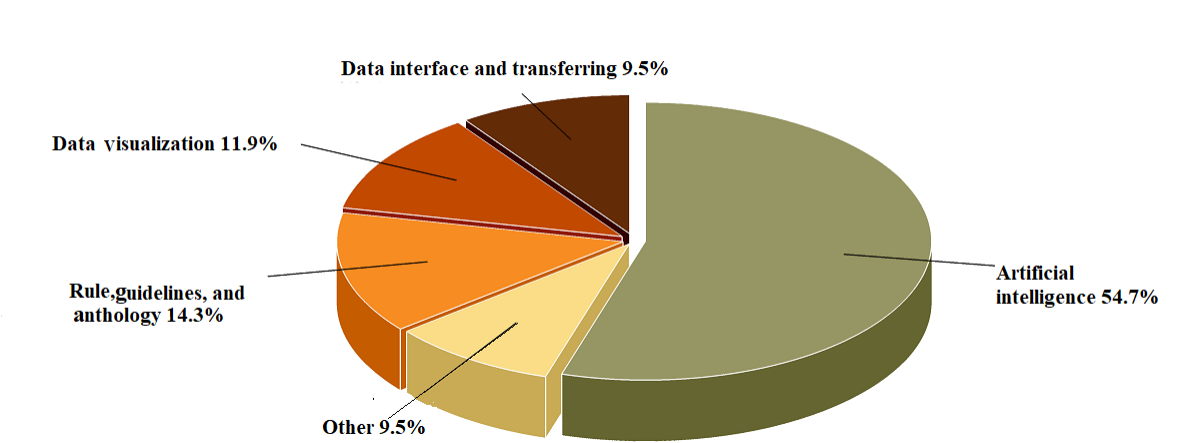
Areas of application of mobile technology for the diagnosis of COVID-19. AI: artificial intelligence.

To answer AQ1, although different apps were used, the main apps belonged to 6 functional domains: contact tracing, data gathering, data visualization, AI-based platform to analyze data and signals, rule- and guideline-based methods for decision-making, and data transformation. Contact tracing is a critical application and a strength of this technology for the timely diagnosis of COVID-19 due to its high availability, serving as a module for accessing GPS satellites, determining people’s positions, and accessing higher data transaction layers, such as cloud and fog spaces. Berquedich et al [35] adopted a model for contact tracing and designed technology to reduce hospital visits and alert people who were in contact with patients to seek health care. Some domains, such as the processing of patients’ voice and audio data for COVID-19 detection, received more attention, whereas biosensor data visualization apps were less frequently designed and presented. Figure 4 depicts the 6 functional dimensions of smartphones for COVID-19 diagnosis.

As can be seen in Figure 4, AI-based methods formed a large part (about 54.7% of the methodologies at the core of smartphones to diagnose COVID-19). It is believed that the researchers’ focus was on mobile phones as on-site processing tools for faster detection of COVID-19. However, the mobile apps that embedded guideline- and rule-based techniques in their processing core to analyze biomarkers are powerful tools for processing all kinds of numerical data and present them to users as a piece of point-of-care equipment. These aspects of mobile apps have been of great importance for researchers due to the ease of faster analysis of input data and the data input gates of mobile phones for more immediate identification of patients. More than 14% of the mobile apps analyzed all kinds of biomarkers using guidelines and rules to access a model in the field of COVID-19 diagnosis by classifying input data. A large number of mobile apps also focused on visualizing (11.9%) and transferring (9.5%) data.

One of the strengths of mobile technology in identifying and diagnosing diseases, especially infectious diseases, is the visualization of the data received from internet of things (IoT) technologies and biosensors. After analyzing the data with hardware chips and equipment or nucleic acid analysis, the signals are transferred to the mobile app for visualization through various means, such as IoT and Bluetooth. In these studies, blood, serology, and saliva data analysis results, after DNA and amino acid analysis, were sent by biosensors to mobile apps for visualization [27,28,47,52].

In some other studies, one of the most influential features of smartphone technology that had a significant impact on COVID-19 diagnosis and detection was the receiving and collection of data from people suspected of having COVID-19. These data included saliva samples, clinical signs and symptoms, vital signs, demographic information, cough sounds, and medical history. The data were obtained with mobile phones, the equipment connected to and embedded in them (eg, chemical sensors), and the recording of vital and clinical signs was stored in the mobile phones or moved to the cloud, allowing other computing devices to access this information. Some studies dealt with using smartphones for collecting patient data; for computation, they provided the data in a centralized space to specialists or decision support systems [21,22,25,26,32,53].

**Figure 5:**
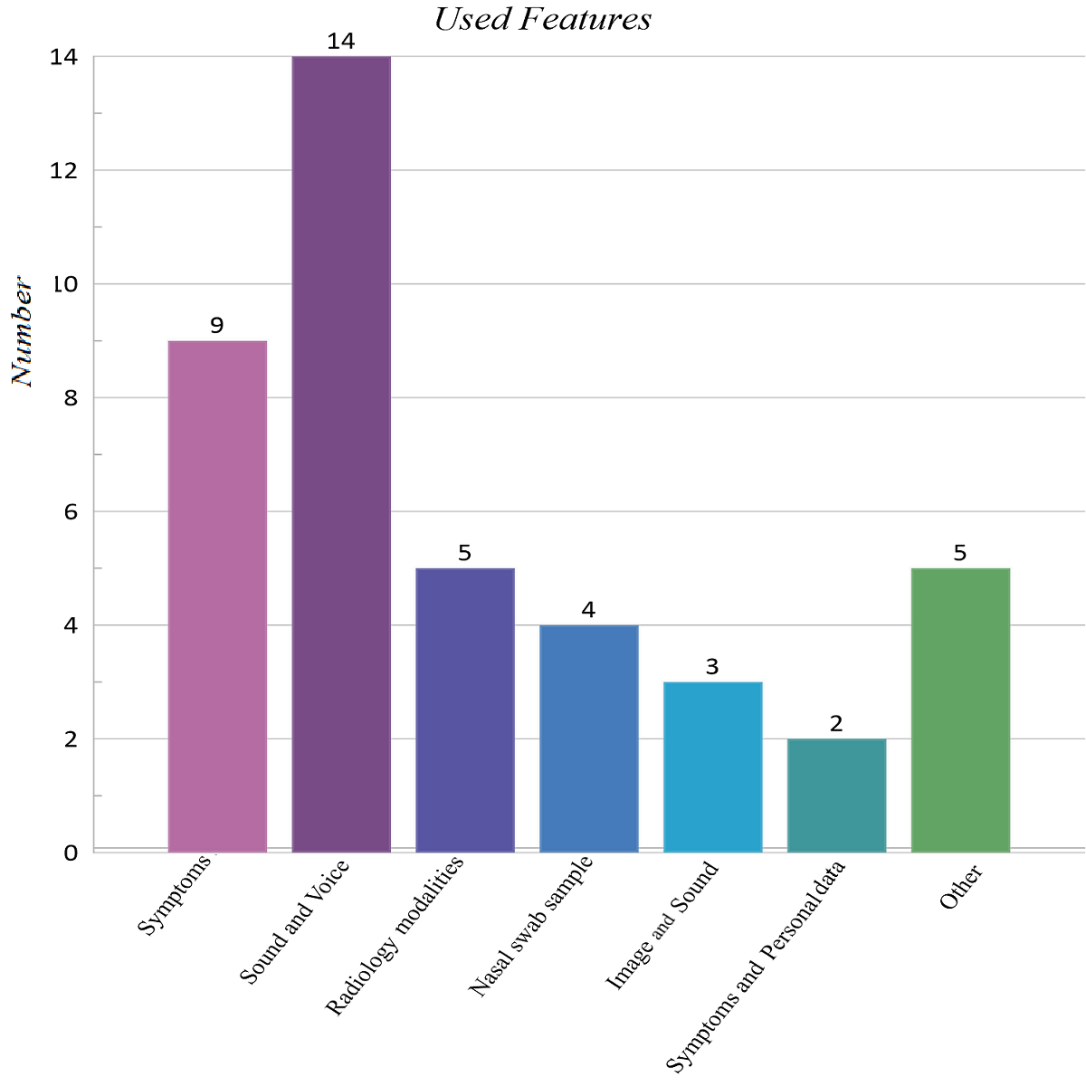
Data used in the detection and diagnosis of COVID-19.

We observed many features to answer AQ2. The design and development of mobile apps for COVID-19 detection and diagnosis were commensurate with the frequency of COVID-19 diagnosis data. In other words, for frequent data, such as coughs, apps that used this type of data to diagnose COVID-19 were the most frequent. Figure 5 presents the usage of data types in mobile apps.

Several laboratory biomarkers, diagnostic tests, biographical data, histories of diseases, and the voices and cough sounds of patients were used as input features for mobile apps based on methodology. According to Figure 5, it seems that the amount of research using these features has a linear relationship with the diagnostic methods of COVID-19, as most mobile apps used the features of signs and symptoms as well as the patients’ voices and cough sounds. In many apps designed to increase the accuracy of disease diagnosis, a combination of these features was used. Since mobile devices can receive and save sounds, these apps used this feature significantly.

For the analysis of nasal swab samples, apps that used biosensor technology incorporated multidisciplinary knowledge and used additional hardware. Following the sample analysis, the resulting data were transferred to the mobile apps via Wi-Fi or Bluetooth and were then used to automatically diagnose COVID-19. As a notable study, one can mention aptamers as a robust molecular tool for COVID-19 diagnosis [12]. The findings revealed that mobile technologies in pandemic control and prevention were a hot research topic and an exciting and trendy approach.

To answer AQ3, the notable use of smartphone apps in COVID-19 prevention assisted clinicians with the timely diagnosis of COVID-19. AI methods, such as ML and DL algorithms, achieved remarkable results in COVID-19 diagnosis. Many researchers [8,10,13,27,35] who used clinical data, such as serology data, vital signs, and symptoms, adopted ML algorithms. These features were received from users and applied to the ML model to diagnose COVID-19. Different ML algorithms were used for this purpose.

Since the main symptoms of COVID-19 diagnosis are cough sounds, the type of coughs, and respiratory sounds, many researchers adopted ML methods and algorithms, such as support vector machines, to identify patients’ sound patterns [10,20,40,48]. Sound feature extraction was carried out based on mathematical algorithms. Using mobile apps to classify these features led to the automatic diagnosis of COVID-19.

Due to the complexity of detecting the pattern of cough sounds, several of the studies required more efficient and voluminous methods, so they used DL algorithms. After training with more significant data and obtaining a more efficient model on systems with more powerful processing units, such as graphics processing units (GPUs), these algorithms were applied to smartphone operating systems with the cough sounds of people suspected of having COVID-19. The methods in this category differentiated infected from noninfected cases by receiving patients’ sounds with higher accuracy and precision [9,30]. Tawfik et al [38] used ML and DL methods simultaneously and achieved 98% accuracy.

Radiographic images offered another facility for rapid diagnosis of COVID-19 and, thus, pandemic control. DL methods and the convolutional neural network (CNN) algorithm were also used in studies using these images in mobile technologies. These models were optimally designed by training on radiology images using concepts such as transfer learning. At the point of care (PoC), they helped clinicians diagnose and detect new cases. These mobile apps demonstrated optimal performance and achieved an accuracy of >92%. By automatically extracting the features using manual feature selection, the bias in the results of diagnostic models was eliminated [18,29,45,46].

AI algorithms were used in various techniques in mobile phones, and the developers attempted to enhance their efficiency by adjusting the parameters of these algorithms [59-62]. Some of the studies used no performance evaluation index to determine the success rate of these apps (to answer AQ4). Regarding apps that used AI methods, the studies used the metrics in the confusion matrix. For different AI methods, different levels of accuracy were obtained. Upon evaluating the efficacy of ML and DL approaches in the identification and diagnosis of COVID-19, our analysis revealed that the accuracy of ML methods ranged from 83% to 99.5%, while DL algorithms exhibited accuracy rates within the range of 91% to 99.6%.

Studies that used all kinds of numerical variables as features, such as signs and symptoms, selected ML methods, while studies that used features such as sounds and images as recognition features, selected DL methods due to their high efficiency.

By calculating the approximate average accuracy of AI-based models in the detection and diagnosis of COVID-19, we concluded that ML algorithms used in mining COVID-19–related data achieved good results. In addition, in analyzing sound data, radiology, or their combination, DL methods achieved high accuracy; in many cases, this accuracy was reported to be >95%. As shown by the accuracy rates presented above, the findings demonstrate the acceptable results of AI methods. In addition, studies using apps to visualize RNA and DNA analysis tests detected cases of COVID-19 in a shorter time due to their sensitivity.

## Discussion

### Principal Findings

In this study, after reviewing all the research conducted on mobile apps for the diagnosis of COVID-19, several research questions that may have arisen for researchers and health care app development companies were answered. This section provides an answer to the remaining AQ by analyzing and evaluating the data entrance, technological method, and performance metrics of the methodologies outlined in the main tables.

**Figure 6:**
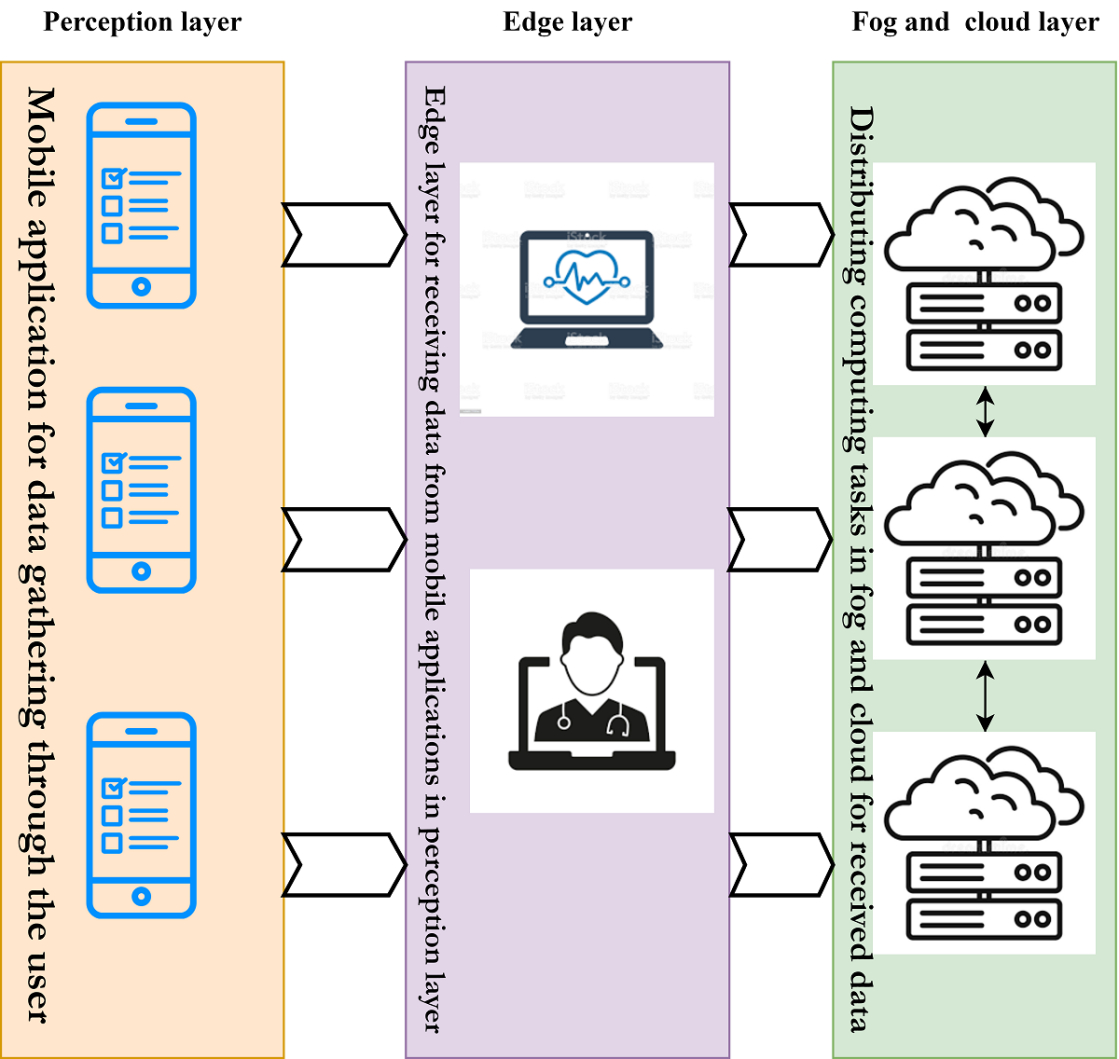
Suggested model for mobile applications for gathering data from smartphones.

To address AQ5, we recommend using mobile apps for gathering data from PoCs obtained from individuals, monitoring devices, or clinical data generators. In modern techniques, edge layer devices are used in the health care devices layer. Therefore, it is strongly advised to incorporate higher-level technologies, such as fog and cloud spaces, for computing processes or computational units in distributed data clusters. Figure 6 illustrates a model of this mobile app for diagnosis.

**Figure 7:**
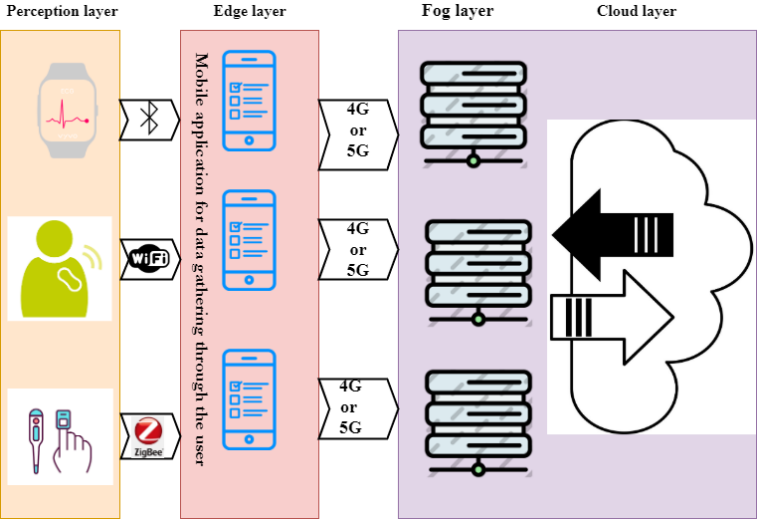
Suggested model for mobile applications for transmitting data from health sensors, biosensors, and monitoring devices. 5G: 5th generation.

In the case of mobile apps that are used as data transmitters at the perception level, we suggest using 5th-generation (5G) instead of 4th-generation (4G) technology in centers and geographical areas that have access to this technology to transfer structured and unstructured data to a higher layer. Using this technology, and transferring health care data to the fog and cloud spaces, distributes computing and data processing. The model in Figure 7 is proposed for mobile apps with the purpose of data collection from sensors, biosensors, and monitoring equipment.

We compared the 42 studies in terms of the requirements for AI methods. In using AI algorithms, the volume of the data set, overfitting prevention methods, and method lightness (for use in mobile phones) are the parameters for analyzing the quality of studies incorporating AI methodology in mobile phones. Since the included studies did not mention these cases, future studies should adopt methods to prevent overfitting when using ML methods. In ML algorithms, it is advised to use 3 methods: early stopping, dropout, and cross-validation. In addition to the mentioned methods, data augmentation techniques should be used in DL methods so that the resulting model is free of any overfitting.

Studies using DL algorithms in smartphones should carefully consider the following points:

- Data preprocessing plays a vital role in model convergence and speed [54]. It is recommended that future studies use data preprocessing techniques, especially for images.
- Model parameters must be optimized to develop a robust and valid model. Future research should adopt the concept of transfer learning and pretrained networks, and these networks should be customized to the investigation.
- Mobile phones have a limited processing unit. As a result, it is suggested that the models be designed using cutting-edge techniques. It is preferred to use lightweight pretrained CNN networks, such as MobileNet, MobileNetV2, and Efficient, to obtain lightweight models that efficiently run on smartphones.
- In studies using mobile phones for data collection and visualization, we recommend using cloud storage and 5G technologies that significantly contribute to the comprehensiveness of the data and image visualization.

### Conclusion

Mobile technology, including various apps, can help with COVID-19 diagnosis and detection and play a vital role in controlling COVID-19 outbreak. Contact tracing can prevent additional contacts during an epidemic or pandemic outbreak of any disease. Thus, on the front lines of outbreak control, healthy people can be separated from people with infection and be alerted through their mobile phones. In the second step, mobile app technology, biosensors (for rapid diagnosis), and AI methods (for diagnosis in the early and acute stages of the disease) can reduce high mortality rates and minimize the consumption of hospital resources. In the third step, mobile technology as a powerful tool can help clinicians form repositories of clinical data and signs and symptoms and collect data from individual smartphones to create such repositories and big data. This can shed more light on COVID-19, its symptoms, the prognosis, and treatment outcomes. In this and future pandemics, smartphones and their apps can be an integral part of controlling the disease and improving patients’ survival.

## Appendix

Multimedia Appendix 1 PRISMA Checklist.

## Abbreviations

5G: 5th generation
AI: artificial intelligence
API: application programming interface
AQ: analytical question
CNN: convolutional neural network
CT: computed tomography
IoT: internet of things
DL: deep learning
ML: machine learning
PCR: polymerase chain reaction
PoC: point of care
PRISMA: Preferred Reporting Items for Systematic Reviews and Meta-Analyses
SpO2: saturation of peripheral oxygen
