# Correction: Mobile Apps for COVID-19 Detection and Diagnosis for Future Pandemic Control: Multidimensional Systematic Review

**DOI:** 10.2196/58810

**Published:** 2024-04-08

**Authors:** Mehdi Gheisari, Mustafa Ghaderzadeh, Huxiong Li, Tania Taami, Christian Fernández-Campusano, Hamidreza Sadeghsalehi, Aaqif Afzaal Abbasi

**Affiliations:** 1 Institute of Artificial Intelligence Shaoxing University Shaoxing China; 2 Department of Computer Science and Engineering, Saveetha School of Engineering Saveetha Institute of Medical and Technical Sciences Chennai India; 3 School of Nursing and Health Sciences of Boukan Urmia University of Medical Sciences Urmia Iran; 4 Florida State University Tallahassee, FL United States; 5 Departamento de Ingeniería Eléctrica Facultad de Ingeniería Universidad de Santiago de Chile Santiago Chile; 6 Department of Neuroscience Faculty of Advanced Technologies in Medicine Tehran Iran; 7 Department of Earth and Marine Sciences University of Palermo Palermo Italy

In “Mobile Apps for COVID-19 Detection and Diagnosis for Future Pandemic Control: Multidimensional Systematic Review” ([JMIR Mhealth Uhealth 2024;12:e44406]) the authors noted one error.

In the original manuscript, the second affiliation for Mehdi Gheisari appeared as follows:

Department of Cognitive Computing, Institute of Computer Science and Engineering, Saveetha School of Engineering, Chennai, India

This has been corrected to:

Department of Computer Science and Engineering, Saveetha School of Engineering, Saveetha Institute of Medical and Technical Sciences, Chennai, India

The correction will appear in the online version of the paper on the JMIR Publications website on April 8, 2024, together with the publication of this correction notice. Because this was made after submission to PubMed, PubMed Central, and other full-text repositories, the corrected article has also been resubmitted to those repositories.

