# Supplementary material for: Mobile Apps for COVID-19 Detection and Diagnosis for Future Pandemic Control: Multidimensional Systematic Review
Source: JMIR Mhealth Uhealth. 2024 Feb 22;12:e44406. doi: 10.2196/44406 (PMC10896318; doi:10.2196/44406)
Supplement: Multimedia Appendix 1 [file mhealth_v12i1e44406_app1.docx]

| **Section and Topic** | **Item #** | **Checklist item** | **Location where item is reported** |
| --- | --- | --- | --- |
| **TITLE** | | |  |
| Title | 1 | Mobile Apps for COVID-19 Detection and Diagnosis for Future Pandemic Control: Multidimensional Systematic Review | 1 |
| **ABSTRACT** | | |  |
| Abstract | 2 | Background: In the modern world, mobile apps are essential for human advancement, and pandemic control is no exception. The use of mobile apps and technology for the detection and diagnosis of COVID-19 has been the subject of numerous investigations, although no thorough analysis of COVID-19 pandemic prevention has been conducted using mobile apps, creating a gap. Objective: With the intention of helping software companies and clinical researchers, this study provides comprehensive information regarding the different fields in which mobile apps were used to diagnose COVID-19 during the pandemic. Methods: In this systematic review, 535 studies were found after searching 5 major research databases (ScienceDirect, Scopus, PubMed, Web of Science, and IEEE). Of these, only 42 (7.9%) studies concerned with diagnosing and detecting COVID-19 were chosen after applying inclusion and exclusion criteria using the PRISMA (Preferred Reporting Items for Systematic Reviews and Meta-Analyses) protocol. Results: Mobile apps were categorized into 6 areas based on the content of these 42 studies: contact tracing, data gathering, data visualization, artificial intelligence (AI)–based diagnosis, rule- and guideline-based diagnosis, and data transformation. Patients with COVID-19 were identified via mobile apps using a variety of clinical, geographic, demographic, radiological, serological, and laboratory data. Most studies concentrated on using AI methods to identify people who might have COVID-19. Additionally, symptoms, cough sounds, and radiological images were used more frequently compared to other data types. Deep learning techniques, such as convolutional neural networks, performed comparatively better in the processing of health care data than other types of AI techniques, which improved the diagnosis of COVID-19. Conclusions: Mobile apps could soon play a significant role as a powerful tool for data collection, epidemic health data analysis, and the early identification of suspected cases. These technologies can work with the internet of things, cloud storage, 5th-generation technology, and cloud computing. Processing pipelines can be moved to mobile device processing cores using new deep learning methods, such as lightweight neural networks. In the event of future pandemics, mobile apps will play a critical role in rapid diagnosis using various image data and clinical symptoms. Consequently, the rapid diagnosis of these diseases can improve the management of their effects and obtain excellent results in treating patients. | 1 |
| **INTRODUCTION** | | |  |
| Rationale | 3 | This study aimed to fill the gap left by previous reviews by conducting a comprehensive review of studies on smartphone apps for the diagnosis of COVID-19, providing solutions based on technological models, and answering research questions so that researchers and health systems can envision devices and their apps in preventing future pandemics. | 3 |
| Objectives | 4 | The main objective was to address the following analytical questions (AQs):  AQ1: What are the uses of smartphones for COVID-19 detection and diagnosis? AQ2: What data do smartphones use to detect and diagnose COVID-19? AQ3: Which artificial intelligence (AI) methods and algorithms are used to process smartphone data? AQ4: How successful have smartphone apps been in COVID-19 detection and classification? AQ5: W hat suggestions can be made to improve the quality of mobile apps in disease diagnosis and pandemic control? |  |
| **METHODS** | | |  |
| Eligibility criteria | 5 | some criteria for inclusion and exclusion of studies. Here is the available information:  **Inclusion criteria:**  Published after the emerging COVID-19.  Studies about the detection and diagnosis of COVID-19.  Meeting existing quality aspects of publication.  Peer-reviewed.  **Exclusion criteria:**  Studies that were not about COVID-19.  Studies not in English context.  Studies not meeting the basic criteria for entry into the research. |  |
| Information sources | 6 | 1. ScienceDirect: A major research database. 2. Scopus: A major research database. 3. PubMed: A major biomedical literature database. 4. Web of Science: A multidisciplinary citation database. 5. IEEE: An online repository for scientific and technical content |  |
| Search strategy | 7 | Our review encompassed the period from November 1, 2019, to late April 2022, extracting pertinent publications that address the intersection of medicine and computer science. By employing the search criteria (("COVID-19") AND (Detection OR Diagnosis) AND (Smartphone OR Mobile Application OR Mobile App)), we ensured a focused approach to gather relevant insights. Notably, the Embase database was omitted from our analysis due to the temporal proximity of publications. |  |
| Selection process | 8 | Relevant studies and the main elements of their methodology and results were recorded in data extraction forms in order to identify AI algorithms and techniques. Two researchers (authors AMR and MG) performed data extraction, and discrepancies between the researchers were resolved by discussion with an independent researcher (author AH). The extracted data elements included the first author’s name, country of origin, research population, data used, purpose, method, the role of the mobile app, and the evaluation method. The search in reputable databases was performed based on the search strategy, and 535 papers were extracted. After reviewing the papers’ abstracts and full texts, applying the inclusion and exclusion criteria, and selecting papers relevant to the title of this study, 42 (7.9%) full-text papers were finally selected. This process was performed based on the PRISMA (Preferred Reporting Items for Systematic Reviews and Meta-Analyses) flowchart. |  |
| Data collection process | 9 | Through this comprehensive examination, we identified PubMed, Web of Science (WoS), Scopus, IEEE, and ScienceDirect as repositories housing the most germane literature. This amalgamation of resources facilitated a robust evaluation of the multifaceted dimensions surrounding the utilization of smartphones and mobile applications in combating the COVID-19 pandemic. |  |
| Data items | 10a | Not Applicable | Not Applicable |
|  | 10b | Not Applicable | Not Applicable |
| Study risk of bias assessment | 11 | Not Applicable | Not Applicable |
| Effect measures | 12 | Not Applicable |  |
| Synthesis methods | 13a | Due to the newly emergent status of COVID-19, the titles, abstracts, and keywords of all the papers published between 2020 and 2022 were reviewed, and 42 (7.9%) of 535 papers were ultimately selected as eligible. By comprehensively examining the mobile apps, we found that the role of smartphones was described in 6 areas with different types of COVID-19 data sets, including “Smartphones play the role of a platform for data collection,” “visualizing the input data,” “installing AI-based processing software,” “determining contact tracing,” and “COVID-19 data processing based on role-based and guideline-based methods,” to detect and diagnose COVID-19. | 4 |
|  | 13b | Not Applicable | Not Applicable |
|  | 13c | In this study, statistical comparisons methods including excel were conducted, and the results were visualized through charts to illustrate the frequency ratio of different methods and items. | 3-8 |
|  | 13d | Not Applicable | Not Applicable |
|  | 13e | Not Applicable |  |
|  | 13f | The sensitivity and other performance indicators were derived by averaging the corresponding values from the research articles included in the study. These averaged values are then presented in Tables 2 and 3 to provide a comprehensive overview of the performance of the evaluated methods or interventions. | 7-8 |
| Reporting bias assessment | 14 | Not Applicable | Not Applicable |
| Certainty assessment | 15 | Not Applicable | Not Applicable |
| **RESULTS** | | |  |
| Study selection | 16a | We reviewed electronic databases publishing papers on medicine and computer science. We concluded that PubMed, Web of Science (WoS), Scopus, IEEE, and ScienceDirect contain the most relevant papers. The search used the following keywords and logical expressions: ((“COVID-19”) AND (Detection OR Diagnosis) AND (Smartphone OR Mobile Application OR Mobile App)). The investigation was conducted from November 1, 2019, to late April 2022, and relevant published papers were extracted. The Embase database was eliminated from the examination due to the proximity of the publications. |  |
|  | 16b | Not Applicable | Not Applicable |
| Study characteristics | 17 | Were about diagnosis and detection of COVID-19 |  |
| Risk of bias in studies | 18 | Not Applicable | Not Applicable |
| Results of individual studies | 19 | Not Applicable | Not Applicable |
| Results of syntheses | 20a | Not Applicable | Not Applicable |
|  | 20b | Not Applicable | Not Applicable |
|  | 20c | Not Applicable | Not Applicable |
|  | 20d | Not Applicable | Not Applicable |
| Reporting biases | 21 | Not Applicable | Not Applicable |
| Certainty of evidence | 22 | Not Applicable | Not Applicable |
| **DISCUSSION** | | |  |
| Discussion | 23a | The 42 studies analyzed in this review categorized mobile apps into six key areas: contact tracing, data gathering, data visualization, AI-based diagnosis, rule- and guideline-based diagnosis, and data transformation. These apps were utilized to identify patients with COVID-19 through a range of clinical, geographic, demographic, radiological, serological, and laboratory data. Predominantly, the focus of these studies was on employing AI methods for the identification of potential COVID-19 cases. Among the various data types utilized, symptoms, cough sounds, and radiological images were particularly prominent. Notably, deep learning techniques, such as convolutional neural networks, exhibited superior performance in processing healthcare data compared to other AI methods, thereby enhancing the accuracy of COVID-19 diagnosis. | 6-9 |
|  | 23b | Discuss any limitations of the evidence included in the review. |  |
|  | 23c | one potential limitation could be the heterogeneity of the studies reviewed, which may impact the generalizability of the recommendations. |  |
|  | 23d | For practice: Healthcare practitioners can consider integrating mobile apps with advanced technological features for data collection and transmission, especially in the context of COVID-19 diagnosis and monitoring. For policy: Policymakers may need to consider promoting the adoption of 5G technology and supporting the development of AI-driven healthcare solutions while ensuring data privacy and security. For future research: There is a need for further research to validate the effectiveness and efficiency of the recommended approaches, especially in real-world settings. Additionally, future studies could explore the integration of emerging technologies like edge computing and blockchain for enhancing the reliability and security of healthcare data transmission and processing. |  |
| **OTHER INFORMATION** | | |  |
| Registration and protocol | 24a | Not Applicable | Not Applicable |
|  | 24b | Not Applicable | Not Applicable |
|  | 24c | Not Applicable | Not Applicable |
| Support | 25 | Not Applicable | Not Applicable |
| Competing interests | 26 | Not Applicable | Not Applicable |
| Availability of data, code and other materials | 27 | Not Applicable | Not Applicable |

*From:*  Page MJ, McKenzie JE, Bossuyt PM, Boutron I, Hoffmann TC, Mulrow CD, et al. The PRISMA 2020 statement: an updated guideline for reporting systematic reviews. BMJ 2021;372:n71. doi: 10.1136/bmj.n71

For more information, visit: <http://www.prisma-statement.org/>
